# Acute kidney injury in patients with myocardial infarction undergoing percutaneous coronary intervention using radial versus femoral access

**DOI:** 10.1186/s12882-019-1210-8

**Published:** 2019-01-30

**Authors:** Vojko Kanic, Gregor Kompara, David Šuran, Alojz Tapajner, Franjo Husam Naji, Andreja Sinkovic

**Affiliations:** 1 Division of Internal Medicine, Department of Cardiology and Angiology, University Medical Center Maribor, Ljubljanska ulica 5, 2000 Maribor, Slovenia; 2 University of Maribor, Faculty of Medicine, Maribor, Slovenia

## Abstract

**Background:**

Data on radial access (RA) as an independent risk factor for acute kidney injury (AKI) in myocardial infarction (MI) patients are conflicting. Our aim was to assess how RA influences the incidence of AKI in MI patients undergoing percutaneous coronary intervention (PCI).

**Methods:**

Data from 3842 MI patients undergoing PCI at our institution from January 2011 to December 2016, of which 35.8% were performed radially, were retrospectively analyzed. A propensity-matched analysis was performed to adjust for differences in the baseline characteristics between the RA and femoral access (FA) groups. The effect of RA on the incidence of AKI was observed.

**Results:**

In the unmatched cohort, AKI occurred less often in the RA group [77 (5.6%) patients in the RA group compared to 250 (10.1%) patients in the FA group; *p* = 0.001]. After propensity-matched adjustment, the incidence of AKI was similar in the two groups.

After adjustment for potential confounders, RA was not identified as an independent predictive factor for AKI in either the unmatched or the propensity-matched cohort. Bleeding, heart failure, age ≥ 70 years, renal dysfunction, and the contrast volume/GFR ratio predicted AKI in both cohorts. Additionally, diabetes, contrast volume, and hypertension were predictive of AKI in the unmatched cohort.

**Conclusion:**

The access site was not independently associated with the incidence of AKI in patients with MI in both a non-matched and a propensity-matched cohort. Our study result suggests that the lower incidence of AKI in patients treated with RA in an unmatched cohort might be substantially influenced by confounding factors, especially bleeding.

**Electronic supplementary material:**

The online version of this article (10.1186/s12882-019-1210-8) contains supplementary material, which is available to authorized users.

## Background

Acute kidney injury (AKI) after percutaneous coronary intervention (PCI) is a well-known complication that is associated with a worse outcome [1–4]. In elective PCI, the contrast volume is presumed to be the major cause of AKI due to its toxic effects [2, 4]. Patients with myocardial infarction (MI) usually require a larger amount of contrast and have a 2.6-fold higher risk of developing AKI [4–7]. Hemodynamic instability, bleeding complications, drug toxicity, and atheroembolism further increase the possibility of AKI in these patients [4, 8]. Data on the association between the access site and the risk of AKI are limited and conflicting. In some reports, the access site was independently associated with AKI [1, 2, 9], but others did not find such an association [10, 11]. Our aim was to assess the possible impact of the access site on the incidence of AKI in patients with MI who had undergone PCI. The group with radial access (RA) was compared with the group with femoral access (FA).

## Methods

The present study is a retrospective single-center analysis of 4082 consecutive patients with MI who underwent PCI. Patients were treated between January 2011 and December 2016 at the University Medical Center Maribor, a tertiary referral hospital with a 24/7 PCI service.

**Fig. 1:**
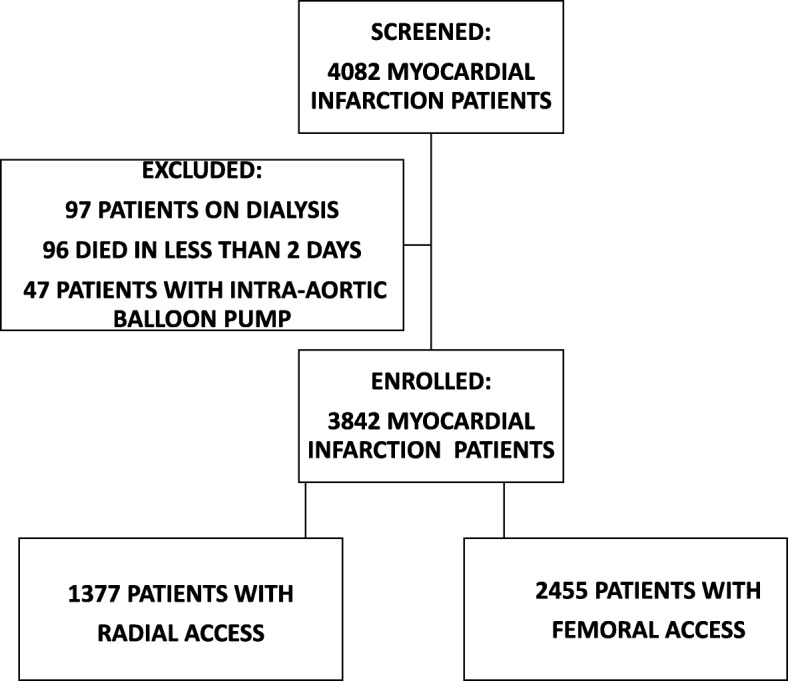
Patient selection. The diagram shows the number of MI patients screened, excluded, and enrolled in the study

We excluded patients on dialysis [97 (1.5%)], patients who required intra-aortic balloon pump implantation [47 (1.1%)], and patients who died in less than 2 days [96 (2.4%)], the assumption being made that there was no time for AKI to occur, leaving 3842 (94.1%) for further analysis (Fig. 1). Thrombolysis was not used.

Patient treatment was based on the current guidelines [12, 13]. The operator decided on the access site, PCI strategy, and concomitant medication. A continuous saline infusion (1 ml/kg/h during PCI and over the next 12 h) was given for renal protection. The hydration rate was reduced at the discretion of the operator and/or attending physician in patients with overt heart failure.

The nonionic contrast agent iopamidol (concentration 370 mgI/ml—Iopamiro 370R, Bracco) was used in our laboratory. Information on the contrast volume given was available for 3729 (97.5%) patients. The study was approved by the Republic of Slovenia Committee for Medical Ethics (No. 40/06/09), which also granted a waiver of consent for this analysis.

### Definitions

The definition of MI was based on the current guidelines [12, 13]. We defined AKI (0.3) as an increase in serum creatinine after PCI of ≥0.3 mg/dl (26.5 μmol/L) in the first 48 h after PCI [14]. AKI (05) was defined as an increase in serum creatinine after PCI ≥ 0.5 mg/dl occurring within the first 48 h after PCI [15]. AKI was also determined using the “Kidney Disease Improving Global Outcomes (KDIGO)” criteria and staged for severity [14].

Heart failure was defined according to clinical criteria (cardiogenic shock or bilateral pulmonary rales, S_3_ gallop, edema) and/or ejection fraction < 30% and/or pulmonary edema on chest X-ray. The ventricular ejection fraction was assessed by bedside echocardiography in the first 48 h after admission.

Anemia was defined as proposed by the World Health Organization: a serum hemoglobin level < 130 g/L for men and < 120 g/L for women [15]. Renal dysfunction on admission was defined as a glomerular filtration rate (GFR) less than 60 ml/kg/1.73m^2^, which was calculated using the 4-variable Modification of Diet in Renal Disease formula from the first blood sample taken on admission [16].

We used the Bleeding Academic Research Consortium (BARC) bleeding criteria and BARC 3a bleeding (Hb drop of 30–50 g/L or any transfusion) was used for calculations [17].

### Outcome

The end point was the incidence of AKI.

### Statistical methods

All data were summarized and displayed as the mean (± standard deviation) or median (25th, 75th percentile) for continuous variables and as the number (percentage) of patients in each group for categorical variables.

The distribution of continuous variables in the RA and FA groups were compared with either the 2-sample *t-*test or the Mann-Whitney test according to whether data followed the normal distribution. Distributions of categorical variables were compared with the chi-square test.

We used binary logistic regression and propensity analysis to determine the association between the vascular access site and AKI in both groups.

The greedy matching technique was used to match an RA patient to an FA patient with the nearest propensity score to permit comparison between RA and FA patients with similar characteristics [18]. The filter included age, gender, ST-elevation MI, PCI of the left main coronary artery, anemia on admission, renal dysfunction on admission, P2Y12 receptor antagonists, contrast volume, and hyperlipidemia. We used the IBM SPSS algorithm, which applies the greedy matching technique to order and sequentially match observations to the nearest propensity score until no matches are possible (propensity scores differ above the default threshold - a caliper of 0.15 of the standard deviation of the logit of the propensity score). The propensity-score-matched cohorts comprised 1377 patients with RA and 2465 patients with FA. An FA match was found for 1049 RA patients, while 328 RA patients remained without an appropriate FA match. After propensity matching, the sample comprised 2098 patients (1049 RA and 1049 FA patients).

Binary logistic regression modeling was used to calculate the adjusted odds of AKI. Age, gender, diabetes, hypertension, ST-elevation MI, heart failure, renal dysfunction on admission, bleeding, upper quartile of contrast volume used, contrast volume/GFR ratio, and RA were used in regression analysis. Data were analyzed using the SPSS 22.0 software for Windows (IBM Corp., Armonk, NY). All *p*-values were two-sided; p-values less than 0.05 were considered statistically significant.

## Results

### Descriptive data for patients before PCI

The study population consisted of 3842 patients with MI, 1377 (35.8%) of whom had RA. The RA patient group was less anemic and had less diabetes and renal dysfunction. Fewer patients in this group suffered ST-elevation MI, heart failure, and bleeding after PCI. They also had lower Mehran scores. Pathology of the left main coronary artery and the left anterior descending artery was less frequently present in this group. A larger volume of contrast was used in the RA group. Basic clinical, angiographic, and therapeutic characteristics of patients differed substantially between the groups, as shown in Tables 1 and 2. After propensity matching, there were no longer any apparent differences between the RA and FA groups (see Additional file 1: Table S1 and Additional file 2: Table S2).

**Table 1:** Basic clinical characteristics of patients

	Radial access *N* = 1377	Femoral access *N* = 2465	*p*
Age, years^a^	64.3 (11.7)	64.9 (12.1)	0.17
Male gender, *N* (%)^b^	975 (70.8)	1722 (69.9)	0.55
Diabetes, *N* (%)^b^	281 (20.4)	584 (23.7)	0.02
Hypertension, *N* (%)^b^	820 (59.5)	1392 (56.5)	0.066
Dyslipidemia, *N* (%)^b^	596 (43.3)	1045 (42.4)	0.61
Anemia, *N* (%)^b^	261 (19.0)	750 (30.4)	< 0.0001
Creatinine, mg/dl^a^	0.96 (0.33)	0.99 (0.40)	0.023
Renal dysfunction, *N* (%)^b^	223 (16.2)	491 (19.9)	0.04
ST-elevation MI, *N* (%)^b^	563 (40.9)	1655 (67.1)	< 0.0001
Heart failure, *N* (%)^b^	128 (9.3)	428 (17.4)	< 0.0001
Left ventricle ejection fraction, %^a^	48.1 (5.8)	46.5 (7.6)	< 0.0001
Mehran score^c^	2.0 (1.0, 5.0)	4.0 (1.0, 8.0)	< 0.0001

^a^Mean (standard deviation); comparison made using the t-test.; ^b^Comparison made using the chi-square test; ^c^Median (25th, 75th percentile); comparison made using the Mann-Whitney U test

*N* number

**Table 2:** Procedural characteristics

	Radial access *N* = 1377	Femoral access *N* = 2465	*p*
P2Y12, *N* (%)^b^	1231 (89.4)	2284 (92.7)	0.001
PCI of the left main coronary artery, *N* (%)^b^	23 (1.7)	96 (3.9)	< 0.0001
PCI of the left anterior descending artery, *N* (%)^b^	517 (37.5)	1039 (42.2)	0.006
PCI of the circumflex artery, *N* (%)^b^	337 (24.5)	520 (21.1)	0.017
PCI of the right coronary artery, *N* (%)^b^	390 (28.3)	784 (31.8)	0.026
Multivessel PCI, *N* (%)^b^	198 (14.4)	381 (15.5)	0.40
Conservative treatment, *N* (%)^b^	67 (4.9)	92 (3.7)	0.09
TIMI flow 0/1 after PCI, *N* (%)^b^	78 (5.1)	125 (5.1)	0.45
Contrast volume, ml^c^	171.0 (125.0, 231.0)	150.0 (110.0, 204.0)	< 0.0001
Contrast volume/GFR ratio^a^	2.45 (1.55)	2.33 (1.80)	0.045
BARC 3a bleeding, *N* (%)^b^	43 (3.1)	195 (7.5)	< 0.0001

^a^Mean (standard deviation); comparison made using the t-test.; ^b^Comparison made using the chi-square test; ^c^Median (25th, 75th percentile); comparison made using the Mann-Whitney test

*BARC* Bleeding Academic Research Consortium, *N* number, *PCI* percutaneous coronary intervention, *P2Y12* P2Y12 receptor antagonists, *TIMI flow* TIMI grade flow after PCI = 0/1

### Acute kidney injury

**Fig. 2:**
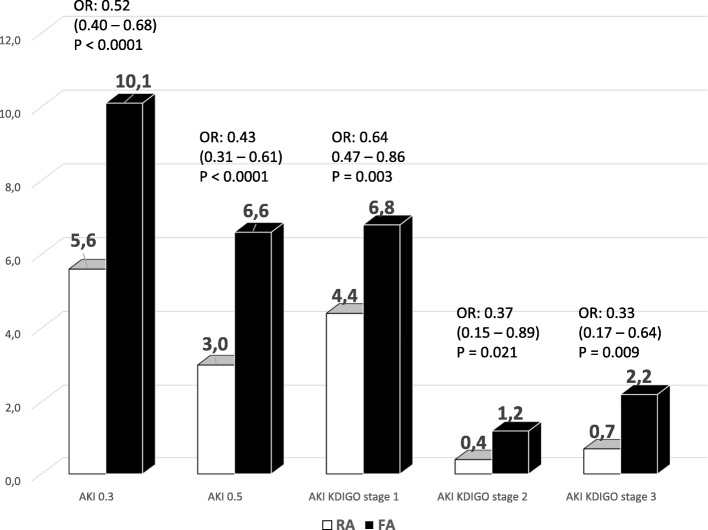
Acute kidney injury in patients with RA and FA with myocardial infarction undergoing PCI in non-matched cohorts. AKI = acute kidney injury; FA = femoral access; KDIGO = Kidney Disease Improving Global Outcomes; OR = odds ratio; RA = radial access

RA patients had a lower unadjusted incidence of AKI (0.3) in the unmatched cohort [77 (5.6%) RA patients suffered AKI (0.3) compared to 250 (10.1%) FA patients; *p* < 0.0001].The unadjusted RA-to-FA odds ratio for AKI was 0.52 (95% confidence interval 0.40 to 0.68; *p* = 0.001). Actually, the prevalence of AKI was lower in RA patients, regardless of which definition of AKI was used (Table 3, Fig. 2).

**Table 3:** Prevalence of AKI in patients with RA and FA according to different definitions

	Radial access *N* = 1377	Femoral access *N* = 2465	All patients *N* = 3842	*p*
AKI (0.3), *N* (%)^a^	77 (5.6)	250 (10.1)	327 (8.5)	< 0.0001
AKI (0.5), *N* (%)^a^	41 (3.0)	163 (6.6)	204 (5.3)	< 0.0001
AKI KDIGO stage 1, *N* (%)^a^	61 (4.4)	167 (6.8)	228 (5.9)	0.003
AKI KDIGO stage 2, *N* (%)^a^	6 (0.4)	29 (1.2)	35 (0.9)	0.021
AKI KDIGO stage 3, *N* (%)^a^	10 (0.7)	54 (2.2)	64 (1.7)	0.001

^a^Comparison made using the chi-square test

*AKI* acute kidney injury, *KDIGO* Kidney Disease: Improving Global Outcomes; *N* number, *RA* radial access, *FA* femoral access

After adjustments for confounders, RA was not identified as an independent predictor of AKI in the unmatched sample (adjusted OR: 0.74; 95% CI: 0.55 to 1.01: *p* = 0.054). Predictors of AKI in the unmatched population are listed in Table 4.

**Table 4:** Independent predictors of AKI in an unmatched and propensity-matched cohort

	Unmatched sample			Propensity score-matched sample		
Variable	OR	95% CI	*p*	OR	95% CI	*p*
Bleeding	4.29	3.07 to 6.00	< 0.0001	5.24	2.42 to 11.37	< 0.0001
Heart failure	3.49	2.64 to 4.61	< 0.0001	2.84	1.72 to 4.69	< 0.0001
Age ≥ 70	2.29	1.72 to 3.05	< 0.0001	2.61	1.65 to 4.13	< 0.0001
Renal dysfunction	1.42	1.01 to 1.98	0.041	1.78	1.02 to 3.11	0.042
Contrast volume /GFR ratio	1.18	1.09 to 1.28	< 0.0001	1.23	1.03 to 1.48	0.021
Diabetes	1.52	1.15 to 2.02	0.004			
Upper quartile of contrast volume	1.45	1.01 to 2.09	0.044			
Hypertension	1.36	1.04 to 1.78	0.023			

*CI* confidence interval, *GFR* glomerular filtration rate, *OR* odds ratio, *AKI* acute kidney injury

After propensity-matched adjustment, the incidence of AKI was similar in the two groups [51 (4.9%) patients in the RA group compared to 65 (6.2%) in the FA group; *p* = 0.21]. Binary logistic regression modeling identified bleeding, heart failure, age ≥ 70 years, renal dysfunction, and the contrast volume/GFR ratio as prognostic factors for AKI (Table 4). However, RA was not found to be a prognostic factor for AKI (adjusted OR: 0.75; 95% CI: 0.50 to 1.11; *p* = 0.15).

## Discussion

We retrospectively analyzed patients with MI who underwent PCI. Patients in the RA group were found to have a lower unadjusted incidence of AKI in the unmatched cohort. However, in a propensity-matched analysis, no difference was found between RA and FA and AKI.

After adjustment for potential confounders, RA was not identified as an independent predictive factor for AKI in either the unmatched or the propensity-matched cohort.

There may be several reasons why the RA group suffered less AKI in the unmatched cohort.

Bleeding, which was less common in the RA group, was the most important predictor of AKI in our analysis. It is known to predict AKI after procedures [13, 19–21], although some data show that RA lowers AKI independently of bleeding [2]. In a prospective trial, RA was not independently associated with AKI when bleeding was entered into the logistic model [20].

ST-elevation MI, which also predicts AKI, was less common in the RA group. Patients with ST-elevation MI present more frequently with hemodynamic impairment (heart failure in 11.2% in ST-elevation MI patients compared to 2.1% in non-ST-elevation MI patients; *p* < 0.0001), which may explain the higher rate of AKI in the FA group [19, 20, 22]. Heart failure, which predisposes to AKI, was less frequent in the RA group [23]. Nevertheless, it is known from previous studies that RA provides consistent benefit across the whole spectrum of patients with ACS [24].

Patients in the RA group were less anemic on admission. Anemia per se has been previously found to be a risk factor for AKI [25]. Moreover, bleeding was seen more often in anemic patients (11.4% vs. 4.3%; *p* < 0.0001), which is in line with previous observations [26]. Patients in the RA group presented with less renal dysfunction on admission. Renal dysfunction is one of the predisposing factors for AKI in MI patients and it also predicted AKI after propensity matching in our analysis [5, 22, 23]. Patients with renal dysfunction also suffered more bleeding (14.0% vs. 4.4%; *p* < 0.0001), as previously reported [13, 27–29].

PCI of the left main coronary artery was less often performed in the RA group. These patients present more frequently with hemodynamic impairment (heart failure was found in 21.6% of patients undergoing PCI of the left main coronary artery compared to 7.2% of patients undergoing non-left main PCI; *p* < 0.0001) and have more complex pathology, which requires a higher contrast volume, as was also found in our study (242.6 ± 105.5 ml vs. 170.0 ± 79.9 ml; *p* < 0.0001) [20]. Anemia (44.5% vs. 25.7%; *p* < 0.0001) and bleeding (12.6% vs. 6.0%; *p* = 0.01) were also more commonly found in these patients.

On the contrary, a higher contrast volume/GFR ratio was found in the RA group. We and others previously have found that the contrast volume/GFR ratio was independently associated with AKI after adjustment for confounders in both the unmatched and the propensity-matched cohort [30–33]. The contrast volume is associated with AKI [1, 20, 34]. However, not all studies showed that the access site contrast volume independently correlated with AKI [10]. The volume of contrast used in our patients and the incidence of AKI (8.5%) were lower than in similar studies (16–22%) [6, 8, 9, 20, 35]. Unfortunately, any comparison is difficult due to differences in patient characteristics and the definition of AKI. Larger contrast volumes were used in RA in our analysis. However, in a similar study of Damluji et al., the contrast volume in the RA group was also larger, and RA was not associated with AKI after propensity matching [10]. Moreover, in MATRIX-AKI, the contrast volume used was similar in both groups, but RA was still not associated with the incidence of AKI when bleeding was taken into account [20]. These data support the finding of Ando et al. that the potential increase in contrast load is unlikely to have any impact on the benefit of RA [1].

In our analysis, bleeding and heart failure influenced AKI more than the contrast volume/GFR ratio. Therefore, despite the higher contrast volume/GFR ratio in RA, less AKI occurred in the RA group in which less bleeding and heart failure occurred.

The strength of the association between RA and AKI varies between studies [2, 10, 11, 20]. The available data suggest that the relationship between RA and AKI might be substantially influenced by confounding factors [1, 2, 10, 11, 20].

The predictors of AKI in our propensity-matched sample were bleeding, heart failure, age ≥ 70 years, renal dysfunction on admission, and the contrast volume/GFR ratio, which is consistent with previously known data [2, 10, 20, 23, 30–32]. These data would seem to support the presumption that AKI, in terms of the access site, might be at least partially influenced by confounding factors. Our result is consistent with the results from the propensity-matched studies of Kolte et al. and Damluji et al. [10, 11].

Our finding has some potential clinical implications. Our data suggest that the better result with RA with reference to AKI is at least partially influenced by confounding factors, most probably bleeding, hemodynamic impairment, age, renal dysfunction on admission, and the contrast volume/GFR ratio. However, in daily practice, RA should be preferred whenever possible. From a clinical point of view, it is irrelevant whether RA is associated with a better outcome per se or if the better outcome is linked to fewer bleeding events and lower rates of other complications due to RA. This is particularly true for patients with a higher bleeding risk (women, older patients, underweight patients, and patients with renal dysfunction) [36]. In addition, early nephroprotective strategies to decrease contrast-induced renal injury, such as low contrast volume, crystalloid infusions, measures to ensure optimal hemodynamics, and discontinuation of nephrotoxic drugs, might provide a significant long-term benefit [27]. It is important to note that radial PCI in patients with MI should only be performed by an experienced radial operator [12, 13].

In conclusion, our data suggest that the lower incidence of AKI in RA patients might be at least partially influenced by confounding factors.

### Limitations

This was an observational and a single-center study. The data about blood pressure and Killip class on admission and evidence-based medical therapy (except for P2Y12 receptor antagonists) after PCI were not available for a sufficient number of patients for these factors to be considered in the evaluation. Other limitations of our study are that the propensity-matching technique cannot adjust for residual confounders and that the baseline number of subjects in each group is somewhat unbalanced, suggesting that the shift from FA to RA has probably happened only recently. Furthermore, we were unable to determine when the shift from FA to RA actually occurred because this has almost certainly been a progressive transition. In previous years, operators were more likely to select less impaired patients for RA, as shown by the lower prevalence of heart failure, anemia, ST- elevation MI patients, and PCI of the left main and left anterior descending arteries in the RA group. Therefore, the results might be different if the propensity-matched population was larger.

## Conclusion

Patients with RA suffered less AKI. However, the access site was not independently associated with a lower incidence of AKI in patients with MI in both non-matched and propensity-matched cohorts. Our study result suggests that the lower incidence of AKI in patients treated with RA in an unmatched cohort might be at least partially influenced by confounding factors, particularly bleeding.

## Additional files

Additional file 1: **Table S1.** Baseline characteristics after propensity matching for the radial access and femoral access groups. (DOCX 13 kb)

Additional file 2: **Table S2.** Procedural characteristics after propensity matching for the RA and FA groups. (DOCX 18 kb)

## Abbreviations

AKI: acute kidney injury
BARC: Bleeding Academic Research Consortium
FA: femoral access
GFR: glomerular filtration rate
KDIGO: Kidney Disease Improving Global Outcomes
LAD: left anterior descending artery
LCX: circumflex artery
LMCA: left main coronary artery
MI: myocardial infarction
N: number
P2Y12: P2Y12 receptor inhibitors
PCI: percutaneous coronary intervention
RA: radial access
RCA: right coronary artery
TIMI: Thrombolysis In Myocardial Infarction

## References

[1] Andò G, Costa F, Trio O, Oreto G, Valgimigli M (2016). Impact of vascular access on acute kidney injury after percutaneous coronary intervention. Cardiovasc Revasc Med.

[2] Kooiman J, Seth M, Dixon S, Wohns D, LaLonde T, Rao SV (2014). Risk of acute kidney injury after percutaneous coronary interventions using radial versus femoral vascular access: insights from the blue cross blue shield of Michigan cardiovascular consortium. Circ Cardiovasc Interv.

[3] Marenzi G, Cosentino N, Moltrasio M, Rubino M, Crimi G, Buratti S, et al. Acute Kidney Injury Definition and In-Hospital Mortality in Patients Undergoing Primary Percutaneous Coronary Intervention for ST-Segment Elevation Myocardial Infarction. J Am Heart Assoc 2016;5:pii: e003522. 10.1161/JAHA.116.003522 PMC5015390 27385429

[4] Azzalini L, Spagnoli V, Ly HQ (2016). Contrast-induced nephropathy: from pathophysiology to preventive strategies. Can J Cardiol.

[5] Shacham Y, Steinvil A, Arbel Y (2016). Acute kidney injury among ST elevation myocardial infarction patients treated by primary percutaneous coronary intervention: a multifactorial entity. J Nephrol.

[6] Marenzi G, Assanelli E, Campodonico J, Lauri G, Marana I, De Metrio M (2009). Contrast volume during primary percutaneous coronary intervention and subsequent contrast-induced nephropathy and mortality. Ann Intern Med.

[7] Tsai TT, Patel UD, Chang TI, Kennedy KF, Masoudi FA, Matheny ME (2014). Contemporary incidence, predictors, and outcomes of acute kidney injury in patients undergoing percutaneous coronary interventions: insights from the NCDR Cath-PCI registry. JACC Cardiovasc Interv.

[8] Anzai A, Anzai T, Naito K, Kaneko H, Mano Y, Jo Y (2010). Prognostic significance of acute kidney injury after reperfused ST-elevation myocardial infarction: synergistic acceleration of renal dysfunction and left ventricular remodeling. J Card Fail.

[9] Cortese B, Sciahbasi A, Sebik R, Rigattieri S, Alonzo A, Silva-Orrego P (2014). Comparison of risk of acute kidney injury after primary percutaneous coronary interventions with the transradial approach versus the transfemoral approach (from the PRIPITENA urban registry). Am J Cardiol.

[10] Damluji A, Cohen MG, Smairat R, Steckbeck R, Moscucci M, Gilchrist IC (2014). The incidence of acute kidney injury after cardiac catheterization or PCI: a comparison of radial vs. femoral approach. Int J Cardiol.

[11] Kolte D, Spence N, Puthawala M, Hyder O, Tuohy CP, Davidson CB (2016). Association of radial versus femoral access with contrast-induced acute kidney injury in patients undergoing primary percutaneous coronary intervention for ST-elevation myocardial infarction. Cardiovasc Revasc Med.

[12] Ibanez B, James S, Agewall S, Antunes MJ, Bucciarelli-Ducci C, Bueno H (2017). 2017 ESC guidelines for the management of acute myocardial infarction in patients presenting with ST-segment elevation. Eur Heart J.

[13] Roffi M, Patrono C, Collet JP, Mueller C, Valgimigli M, Andreotti F (2016). 2015 ESC guidelines for the management of acute coronary syndromes in patients presenting without persistent ST-segment elevation. Eur Heart J.

[14] Kellum JA, Lamiere J, Aspelin P, Barsoum RS, Burdmann EA, Goldstein SR, et al. KDIGO 15. Solomon R, Dauerman HL Contrast-induced acute kidney injury Circulation 2010;122:2451–5. 10.1161/CIRCULATIONAHA.110.953851 21135373

[15] Nutritional anaemias (1968). Report of a WHO scientific group. Haemoglobin Concentrations for the Diagnosis of Anaemia and Assessment of Severity VMNIS.

[16] Levey AS, Coresh J, Greene T, Marsh J, Stevens LA, Kusek JW (2007). Expressing the modification of diet in renal disease study equation for estimating glomerular filtration rate with standardized serum creatinine values. Clin Chem.

[17] Mehran R, Rao SV, Bhatt DL, Gibson CM, Caixeta A, Eikelboom J (2011). Standardized bleeding definitions for cardiovascular clinical trials: a consensus report from the bleeding academic research consortium. Circulation.

[18] Parsons L (2001). Reducing bias in a propensity score matched-pair sample using greedy matching techniques. Proceedings of the 26th annual SAS users group international conference.

[19] Kindgen-Milles D (2014). Acute kidney injury in the perioperative setting. Med Klin Intensivmed Notfmed.

[20] Andò J, Cortese B, Russo F, Rothenbühler M, Frigoli E, Gargiulo G (2017). Acute kidney injury after radial or femoral access for invasive acute coronary syndrome management. J Am Coll Cardiol.

[21] Valgimigli M, Gagnor A, Calabró P, Frigoli E, Leonardi S, Zaro T (2015). Radial versus femoral access in patients with acute coronary syndromes undergoing invasive management: a randomised multicentre trial. Lancet.

[22] Marenzi G, Cabiati A, Milazzo V, Rubino M (2012). Contrast-induced nephropathy. Intern Emerg Med.

[23] Marenzi G, Cosentino N, Bartorelli AL (2015). Acute kidney injury in patients with acute coronary syndromes. Heart.

[24] Vranckx P, Frigoli E, Rothenbühler M, Tomassini F, Garducci S, Andò G (2017). Radial versus femoral access in patients with acute coronary syndromes with or without ST-segment elevation. Eur Heart J.

[25] Han SS, Baek SH, Ahn SY, Chin HJ, Na KY, Chae DW (2015). Anemia is a risk factor for acute kidney injury and long-term mortality in critically ill patients. Tohoku J Exp Med.

[26] Bassand JP, Afzal R, Eikelboom J, Wallentin L, Peters R, Budaj A (2010). Relationship between baseline haemoglobin and major bleeding complications in acute coronary syndromes. Eur Heart J.

[27] Kanic V, Suran D, Vollrath M, Tapajner A, Kompara G (2017). Influence of minor deterioration of renal function after PCI on outcome in patients with ST-elevation myocardial infarction. J Interv Cardiol.

[28] Franczyk-Skóra B, Gluba A, Banach M, Rozentryt P, Poloński L, Rysz J (2013). Acute coronary syndromes in patients with chronic kidney disease. Curr Vasc Pharmacol.

[29] Saltzman AJ, Stone GW, Claessen BE, Narula A, Leon-Reyes S, Weisz G (2011). Long-term impact of chronic kidney disease in patients with ST-segment elevation myocardial infarction treated with primary percutaneous coronary intervention: the HORIZONS-AMI harmonizing outcomes with revascularization and stents in acute myocardial infarction trial. JACC Cardiovasc Interv.

[30] Mager A, Vaknin Assa H, Lev EI, Bental T, Assali A, Kornowski R (2011). The ratio of contrast volume to glomerular filtration rate predicts outcomes after percutaneous coronary intervention for ST-segment elevation acute myocardial infarction. Catheter Cardiovasc Interv.

[31] Margolis G, Gal-Oz A, Letourneau-Shesaf S, Khoury S, Keren G, Shacham Y. Acute kidney injury based on the KDIGO criteria among ST elevation myocardial infarction patients treated by primary percutaneous intervention. J Nephrol. 2017. 10.1007/s40620-017-0461-3. 10.1007/s40620-017-0461-3 29185210

[32] Celik O, Ozturk D, Akin F, Ayca B, Yalcın AA, Erturk M (2015). Association between contrast media volume–glomerular filtration rate ratio and contrast-induced acute kidney injury after primary percutaneous coronary intervention. Angiology.

[33] Andò G, de Gregorio C, Morabito G, Trio O, Saporito F, Oreto G (2014). Renal function-adjusted contrast volume redefines the baseline estimation of contrast-induced acute kidney injury risk in patients undergoing primary percutaneous coronary intervention. Circ Cardiovasc Interv.

[34] Mehran R, Aymong ED, Nikolsky E, Lasic Z, Iakovou I, Fahy M (2004). A simple risk score for prediction of contrast-induced nephropathy after percutaneous coronary intervention. J Am Coll Cardiol.

[35] Narula A, Mehran R, Weisz G, Dangas GD, Yu J, Généreux P (2014). Contrast-induced acute kidney injury after primary percutaneous coronary intervention: results from the HORIZONS-AMI substudy. Eur Heart J.

[36] Gupta S, Cigarroa JE (2014). Bleeding, a call to action. Catheter Cardiovasc Interv.

