# Supplementary material for: Acute kidney injury in patients with myocardial infarction undergoing percutaneous coronary intervention using radial versus femoral access
Source: BMC Nephrol. 2019 Jan 30;20:28. doi: 10.1186/s12882-019-1210-8 (PMC6354416; doi:10.1186/s12882-019-1210-8)
Supplement: Supplementary file 1 — Table S1. Baseline characteristics after propensity matching for the radial access and femoral access groups. (DOCX 13 kb) [file 12882_2019_1210_MOESM1_ESM.docx]

**Additional file 1: Table S1**

Baseline characteristics after propensity matching for the radial access and femoral access groups

| Propensity-matched sample ^¤^ | | | |
| --- | --- | --- | --- |
|  | Radial access  N = 1049 | Femoral access  N = 1049 | p |
| Age, years∞ | 64.1 (11.6) | 64.1 (11.5) | 0.99 |
| Male gender, N (%)* | 743 (68.8) | 719 (68.5) | 0.27 |
| Diabetes, N (%)* | 222 (21.2) | 242 (23.1) | 0.32 |
| Hypertension, N (%)* | 631 (60.2) | 650 (62.0) | 0.42 |
| Dyslipidemia, N (%)* | 468 (44.6) | 468 (44.6) | 1.00 |
| Anemia, N (%)* | 188 (17.9) | 188 (17.9) | 1.00 |
| Admission creatinine, mg/dl | 0.95 (0.33) | 0.95 (0.30) | 0.62 |
| Renal dysfunction, N (%)* | 169 (16.1) | 167 (15.9) | 0.95 |
| ST-elevation MI, N (%)* | 457 (43.6) | 457 (43.6) | 1.00 |
| Heart failure, N (%)* | 83 (7.9) | 81 (7.7) | 0.93 |
| Left ventricle ejection fraction, %∞ | 48.4 (5.4) | 48.5 (5.3) | 0.87 |
| Mehran score | 2.0 (1.0, 5.0) | 2.0 (1.0, 5.0) | 0.63 |

¤ The propensity-matched sample was balanced by age, gender, ST-elevation MI, PCI of the left main coronary artery, anemia on admission, renal dysfunction on admission, P2Y12 receptor antagonists, contrast volume, and hyperlipidemia.

∞ Mean (standard deviation); comparison made using the t-test; * Comparison made using the chi-square test; ¥ Median (25th, 75th percentile); comparison made using the Mann-Whitney test.

MI = myocardial infarction; N= number.
