# Supplementary material for: Acute kidney injury in patients with myocardial infarction undergoing percutaneous coronary intervention using radial versus femoral access
Source: BMC Nephrol. 2019 Jan 30;20:28. doi: 10.1186/s12882-019-1210-8 (PMC6354416; doi:10.1186/s12882-019-1210-8)
Supplement: Supplementary file 2 — Table S2. Procedural characteristics after propensity matching for the RA and FA groups. (DOCX 18 kb) [file 12882_2019_1210_MOESM2_ESM.docx]

**Additional file 2: Table S2**

Procedural characteristics after propensity matching for the RA and FA groups

| Propensity-matched sample ^¤^ | | | |
| --- | --- | --- | --- |
|  | Radial access  N = 1049 | Femoral access  N = 1049 | p |
| P2Y12, N (%)* | 994 (91.7) | 994 (91.7) | 1.00 |
| PCI of the left main coronary artery, N (%)* | 3 (0.3) | 3 (0.3) | 1.00 |
| PCI of the left anterior descending artery, N (%)* | 364 (34.7) | 364 (34.7) | 1.00 |
| PCI of the circumflex artery, N (%)* | 206 (19.6) | 206 (19.6) | 1.00 |
| PCI of the right coronary artery, N (%)* | 311 (29.6) | 294 (27.1) | 0.21 |
| Conservative treatment, N (%)* | 60 (5.7) | 75 (7.1) | 0.21 |
| TIMI flow 0/1 after PCI, N (%)* | 53 (5.1) | 58 (5.5) | 0.70 |
| Contrast volume, ml^¥^ | 160.0 (120.0, 207.0) | 150.0 (116.5, 200.0) | 0.078 |
| Contrast volume/GFR ratio∞ | 2.18 (1.24) | 2.13 (1.22) | 0.25 |
| Bleeding, N (%)* | 18 (1.7) | 18 (1.7) | 1.00 |

¤ The propensity-matched sample was balanced by age, gender, ST-elevation MI, PCI of the left main coronary artery, anemia on admission, renal dysfunction on admission, P2Y12 receptor antagonists, contrast volume, and hyperlipidemia.

∞ Mean (standard deviation); comparison made using the t-test.; * Comparison made using the chi-square test; ¥ Median (25th, 75th percentile); comparison made using the Mann-Whitney test.

N= number; PCI = percutaneous coronary intervention; P2Y12 = P2Y12 receptor antagonists, TIMI = Thrombolysis In Myocardial Infarction.
